# Remdesivir Efficacy and Tolerability in Children with COVID-19-Associated Allergic Comorbidities such as Asthma, Allergic Rhinitis, and Atopic Dermatitis

**DOI:** 10.3390/children10050810

**Published:** 2023-04-29

**Authors:** Gheorghiță Jugulete, Monica Luminos, Carmen Pavelescu, Mădălina Maria Merișescu

**Affiliations:** 1 Faculty of Medicine, University of Medicine and Pharmacy, “Carol Davila”, No. 37, Dionisie Lupu Street, 2nd District, 020021 Bucharest, Romania; 2 “Matei Balş” National Institute for Infectious Diseases, No. 1, Calistrat Grozovici Street, 2nd District, 021105 Bucharest, Romania

**Keywords:** COVID-19, antiviral therapy, children, SARS-CoV-2, remdesivir, allergies, asthma, atopic dermatitis, allergic rhinitis

## Abstract

In children, coronavirus disease 2019 (COVID-19) starts as a minor illness compared to adults, but during the ongoing COVID-19 pandemic, distinct SARS-CoV-2 variants and subvariants have changed options for therapies in both adults and children, especially for those with comorbidities such as allergies. On 25 April 2022, Remdesivir (RDV), a viral RNA-dependent RNA polymerase inhibitor, was approved by the Food and Drug Administration (FDA) for the treatment of pediatric patients 28 days and older, weighing ≥3 kg, hospitalized or non-hospitalized, who are at high risk of progression to severe forms of COVID-19. While RDV has been shown to have favorable effects in numerous types of research conducted on adults, such as shortening hospital stays, and has shown it has antiviral effects on various RNA viruses, there is a lack of findings regarding safety, tolerability, and efficacy of RDV in allergic pediatric patients since its initial FDA approval. This study aims to assess RDV’s efficacy and tolerability in treating pediatric patients with mild and severe forms of COVID-19-associated allergies such as asthma, allergic rhinitis, and atopic dermatitis and how RDV affects the duration of hospitalization, especially for these comorbidities. The most recent pandemic wave among children rose due to the high transmissibility of the Omicron variant, and this study analyzed changes between July 2020 and September 2022 at the National Institute of Infectious Diseases “Prof. Dr. Matei Balș”, Bucharest, Romania. Our retrospective study included 250 children <18 years old, 42 (16.8%) had allergies, 132 were males (52.8%), age group 0–5 years old (80%), with a positive viral test for SARS-CoV-2. Severity was categorized as mild (43.6%), moderate (53.2%), and severe (1.6%) COVID-19, and treatment with RDV was administered in 50.4% (126/250) of children included in the study. The presence of comorbidities, asthma (7.2%), allergic rhinitis (4.4%), and atopic dermatitis (4.4%), was associated with an increased risk of developing severe COVID-19 infection in children, *p* < 0.05. We did not register deaths and severe complications; all cases evolved favorably under the instituted treatment. Laboratory abnormalities in transaminase levels 53.97% (ALT) and 61.9% (AST) were grades 1 or 2 and did not require discontinuation of the antiviral treatment, *p* < 0.05. RDV in children reduced the duration and evolution of COVID-19 and decreased the length of hospitalization in group-associated allergies; *p* < 0.05. This article summarizes RDV’s efficacy among children with COVID-19 and allergies when the clinical result was improved and reports positive effects on tolerability and reduced duration of hospitalization, especially in children with asthma, atopic dermatitis, and allergic rhinitis. More studies are needed to confirm our findings.

## 1. Introduction

**Figure 1:**
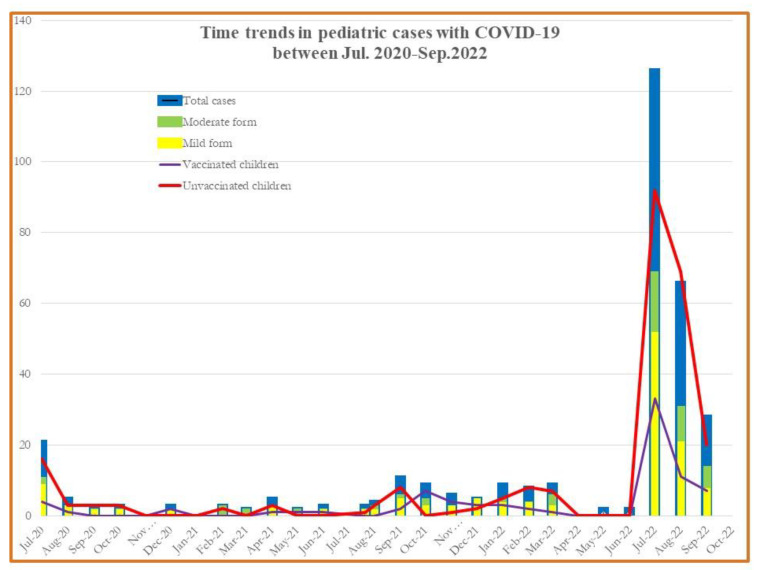
Monthly COVID-19 case incidence among patients aged <18 years old, hospitalized between July 2020–September 2022.

Since the COVID-19 pandemic was initially reported in March 2020, it has been demonstrated that children have a much lower incidence of SARS-CoV-2 infection than adults and most infected children either experience mild sickness or no symptoms at all [1,2,3,4]. The epidemiology of COVID-19 in pediatric patients has undergone a significant change because of the pandemic’s spread, the use of COVID-19 vaccines among adults, and the appearance of SARS-CoV-2 variants. The incidence of pediatric COVID-19 infection requiring hospitalization increased worldwide [5,6,7,8]. In Romania, the prevalence of pediatric COVID-19 cases within the overall number of COVID-19 cases diagnosed between 1 July 2020 and 30 September 2022 had its peak hospitalization rate during the final pandemic months (See Figure 1). The peak during the Omicron variant was roughly higher than the peak during the previous Delta variant period, with the biggest differences occurring in infants [9,10]. 

The results of the pollen monitoring in Bucharest during the previous years revealed a sizable number of allergenic pollen. Pollen levels in Bucharest’s atmosphere grew from May to September, with peaks in June, July, and September as well, indicating a longer period than previously thought. Relevant observations included the seasonal new case rate increasing by 2.5–3 times over the past few years, with notable increases in the most recent year. Nearly all allergists indicated that 2–5 new verified cases were recorded each day. Seasonal allergic rhinitis, or rhino-conjunctivitis, was the most prevalent clinical kind of allergy, and 20% of patients had also recently developed asthma. Up to 90% of pediatric asthma cases have an allergic etiology, and children who have allergies have a 30% higher chance of getting asthma [11]. 

Of the many recognized causes of asthma flare-ups and poorly managed asthma, respiratory viruses are the most clearly described. SARS-CoV-2 and other respiratory viral infections such as human rhinovirus, respiratory syncytial virus, and influenza virus are significant causes of morbidity and asthma exacerbations [12]. To date, various studies have reevaluated the significance of allergic sensitization concerning COVID-19, no longer viewing it as a risk factor but rather as a protective one against SARS-CoV2 infection [12,13,14,15,16].

As an antiviral medication, RDV is a nucleotide analogue that specifically inhibits the SARS-CoV-2 RdRP as well as the RdRP of other viruses and has been administered in children after the drug met the condition of approval. RDV is an antiviral drug developed by Gilead Sciences, and its primary prescription was for the Ebola virus infection with poor efficacy in a clinical trial of phase III [17,18].

Remdesivir, as an intravenous nucleotide prodrug that binds to viral RNA-dependent RNA polymerase and prevents viral replication, is the only antiviral medication against COVID-19 that has received approval to date [17,18,19,20,21,22]. This medication was found to have a faster recovery time than a placebo in hospitalized people who had signs of a lower respiratory infection and needed extra oxygen. As a result, it is necessary to treat early disease stages with additional antiviral medications to avoid hospitalization and deaths brought on by disease development [19,22,23,24,25]. RDV was first approved by the FDA in October 2020 to treat adults and pediatric patients 12 years of age and older and weighing at least 40 kg with severe COVID-19 that required hospitalization. RDV was authorized by the FDA in April 2022 for the treatment of pediatric patients who are hospitalized or not and who are over 28 days old and weighing at least 3 kg and who are at high risk of progressing to severe COVID-19, including hospitalization, since its effectiveness has been reported in several observational studies [26,27]. The lyophilized formulation is preferred for pediatric patients weighing more than 3.5 kg but less than 40 kg. On day 1, a single loading dosage of 5 mg/kg of body weight should be given, followed by a 2.5 mg/kg maintenance dose, which should be injected intravenously to achieve the best concentration. Interactions of RDV with other drugs in humans have not been reported, and the benefits were better when it was administered earlier in the disease. If patients do not show clinical improvement after 5 days of treatment, the duration of treatment may be increased to 10 days for pediatric patients with moderate or severe COVID-19 [27,28,29].

Thus, we aimed to investigate the efficacy and safety of Remdesivir therapy in COVID-19 pediatric patients and how intravenous RDV reduced time to hospital recovery and improved their clinical status, especially in patients with allergies such as asthma, allergic rhinitis, and atopic dermatitis.

## 2. Methods

A retrospective study was conducted at the Pediatric Clinical Section IX of the National Institute of Infectious Diseases “Prof. Dr. Matei Balș”, Bucharest, Romania, after approval from the institutional ethical review committee, C0033/10.01.2023. Consent was obtained from the patients and their parents as per the requirements of the ethics boards. The study included 250 pediatric patients aged <18 years old, admitted with COVID-19 in Clinical Section IX from July 2020 to September 2022. Nasopharyngeal swabs were taken from all patients who presented an acute illness at the National Institute for Infectious Diseases, Bucharest, Romania within 24 h of admission and tested using a reverse transcription-polymerase chain reaction (RT-PCR) kit (Thermo Scientific). We included pediatric patients eligible for a RDV regimen (intravenous) with allergies and compared them with the group with allergies on a standard regimen, except RDV. For children under 40 kg, a single dose of 5 mg/kg on day one was prescribed, followed by a daily dose of 2.5 mg/kg from day 2 up to 10 days of hospitalization. Children who were 40 kg or more at screening received a single 200 mg dose on day one, followed by a daily 100 mg dose for the next days (2–10). The severity of the disease was classified according to the national guidelines into mild, moderate, and severe disease. 

Allergic symptoms for all the children with allergies included in our study were evaluated and diagnosed in the past by specialist physicians. Skin prick testing, specific immunoglobulin E (IgE) levels, and spirometry results were completed in the medical record, along with demographic data regarding pediatric patients, comorbidities, clinical presentation, and laboratory parameters including details of infections, and the outcomes were saved in the medical records. 

The diagnosis of asthma in the study participants was collected from patient records, consisting of demographic data, asthma-related clinical history (medications used, visits to the emergency department, hospitalizations and/or intensive care unit admissions for asthma ever), and asthma severity. The history of exacerbations requiring the use of systemic corticosteroids; total IgE levels; eosinophil number; and allergic sensitization by skin prick test and/or specific IgEs to common perennial and seasonal allergens (Dermatophagoides pteronyssinus, Dermatophagoides farinae, trees, grasses and weeds pollens, molds, cat and dog danders); a mixture of grass and cereal pollens, Betulaceae pollen, weed pollen (Artemisia and Ambrosia); Alternaria alternata; cockroaches (Blattella germanica); and a mixture of feathers were all part of the test panel and comorbidity data (allergic rhinitis, atopic dermatitis). Two or more nasal symptoms, such as congestion, rhinorrhea, sneezing, and aeroallergen sensitivity detected on SPT or an allergen-specific IgE, were necessary for the diagnosis of allergic rhinitis (AR). The Hanifin–Rajka criteria were used to make the diagnosis of atopic dermatitis (AD).

Data were analyzed using GraphPad Prism Quantitative variables such as age and length of stay, and values of inflammatory markers were presented as a number and (%), median and interquartile range (IQR). Qualitative variables such as gender, comorbidities, clinical presentation, disease severity, details of infections, and outcomes were presented as median (IQR) depending on the distribution of the data. Univariate and multivariable logistic regression models were used to find independent predictors of mild, severe, or critical COVID-19. A three-way ANOVA test was used before and after RDV administration in children with allergies for clinical outcomes. A *p*-value of <0.05 was taken as statistically significant.

## 3. Results

The monthly COVID-19-associated number of hospitalizations in children and adolescents during the week ending July 2022 was nearly five times (20 vs. 125) the number during the week ending July 2020. Hospitalizations for both vaccinated and unvaccinated children during July 2022–September 2022 for the mild and moderate forms among children aged 0–18 years was nearly 5.23 times compared with a similar period in 2020 (47 vs. 246) and 10 times higher for 2021 (See Figure 1).

A total of 250 pediatric patients were enrolled in this study between 1 July 2020 and 30 September 2022 in our Pediatric Department. The median age of the pediatric population was 3 years old (IQR 2–16). There were 132 males, (52.8%), and 38.8% were in the 0–1 year old age category. In the first group, 126 pediatric patients received RDV and symptomatic therapy and in the second group, 124 pediatric patients received only symptomatic treatment (non-RDV group). While 43.6% of the total group were mild cases, 53.2% of cases had moderate forms of COVID-19 and 1.6% were in a severe category. 

**Figure 2:**
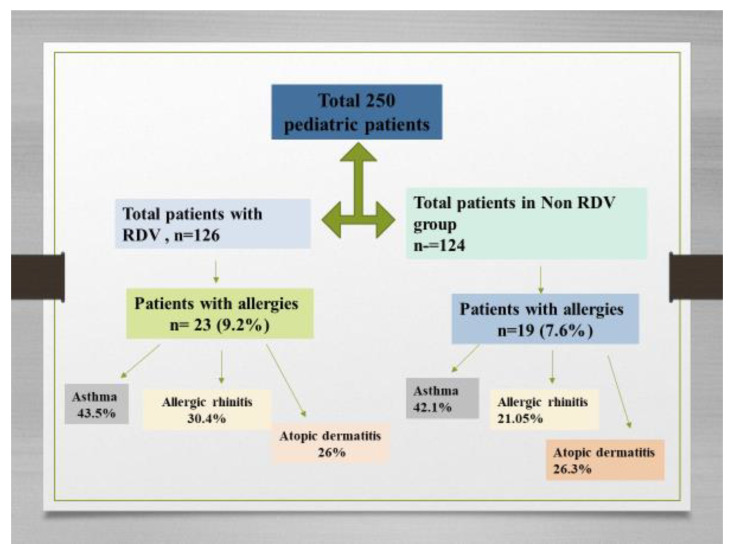
Flow diagram showing the enrollment of patients and allocation to RDV group and non-RDV group and the pediatric patients with allergies.

We performed subgroup analyses by comorbidities and found that the total number of children with allergies was 42 (16.8%). Based on the results of the medical records, in 250 pediatric patients hospitalized with COVID-19, 18 (7.2%) had a history of asthma and 4.4% had atopic dermatitis and allergic rhinitis for each group. Of the 42 pediatric patients with allergies, 23 (9.2%) received RDV and 19 (7.6%) were included in the non-RDV group. Among the 23 children with RDV treatment, 43.5% had asthma (See Figure 2). The results consistently demonstrated differences according to RDV administration for asthma, atopic dermatitis, and allergic rhinitis. The median duration of COVID-19 symptoms was shorter in the group receiving RDV with allergies at baseline (8 days, IQR [5,6,7,8,9,10]) than in the non-RDV group (14 days, IQR [8,9,10,11,12,13,14,15,16]) (*p* < 0.001). The rate of COVID-19 hospital admission was higher in children with poorly controlled asthma than in those with well-controlled asthma or without asthma. All cases progressed favorably; we recorded no deaths. 

We performed a multivariate analysis to assess the association between the demographic characteristics and the clinical outcomes. 

The result of this analysis showed that 88.07% of the total cases had a fever, (>38.5 °C), *p* = 0.001. 67.89% had digestive symptoms (vomiting, diarrhea), and 85.32% had respiratory symptoms, (*p* = 0.001 and 0.05, respectively) (See Table 1). Gastroenteritis and pneumonia occurred in 51.82% and 36.5% of patients with moderate COVID-19 and had significant statistical values, *p* = 0.001 and 0.05. Patients receiving RDV therapy revealed more clinical improvement than those in the non-RDV group [OR = 1.56 (95% CI 1.24–2.83]. 

Comorbidities associations between obesity and asthma and RDV administrations were found to have statistical significance (*p* < 0.001). We observed a lower risk of serious adverse reactions in the RDV group [OR = 0.69 (95% CI 0.45–0.84)] than in the non-RDV group [OR = 0.39 (95% CI 0.25–0.60)]. 

**Figure 3:**
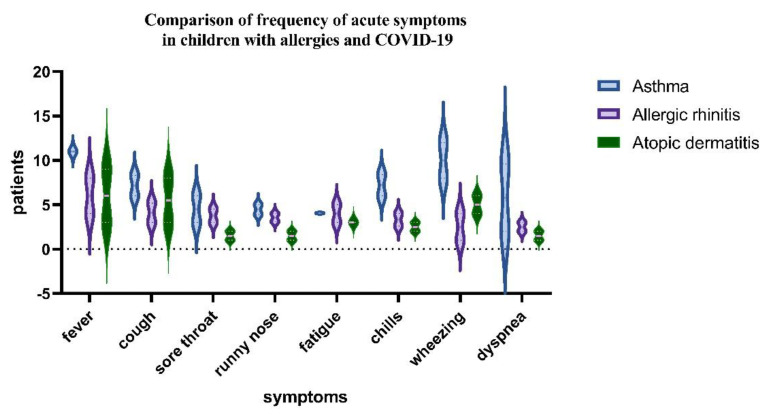
One sample test and Wilcoxon test comparison of the acute symptoms of COVID−19 between asthmatics group, allergic rhinitis, and atopic dermatitis children (*p*−value < 0.05).

In the group of 250 pediatric patients with a positive history of allergies, the following was finally diagnosed: 11 (4.4%) had allergic rhinitis and 11 were found with atopic dermatitis. Eighteen pediatric patients (7.2%) had asthma. We found patients with two comorbidities in 14.8% of cases and 16% with one comorbidity. The most common allergens were weeds pollens (Artemisia and Ambrosia), grass pollen, house dust and flour dust mites, tree pollen, cat and dog allergens, Dermatophagoides pteronyssinus, and Dermatophagoides farinae. There was a correlation between the proportion of diagnoses of asthma and allergic rhinitis and RDV administration. The performed statistical analysis allowed us to observe that patients with allergies and COVID-19 have symptoms such as fever, wheezing, and dyspnea and that these were statistically significant for asthmatic patients. The main symptoms related to asthma-associated COVID-19 are shown in Figure 3.

When adjusted for SpO2, we found statistical improvements in the group with RDV for obesity, asthma, and CPD compared with the non-RDV group (See Table 2).

**Figure 4:**
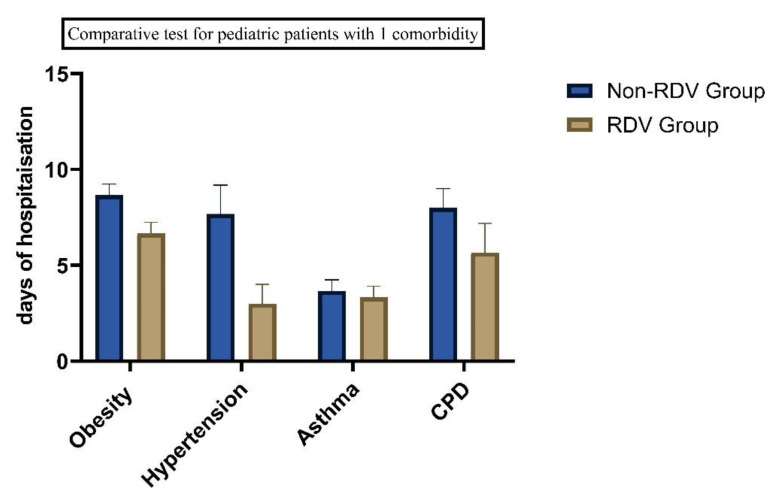
Days of hospitalization in children with COVID-19 and 1 comorbidity (*p* < 0.05).

**Figure 5:**
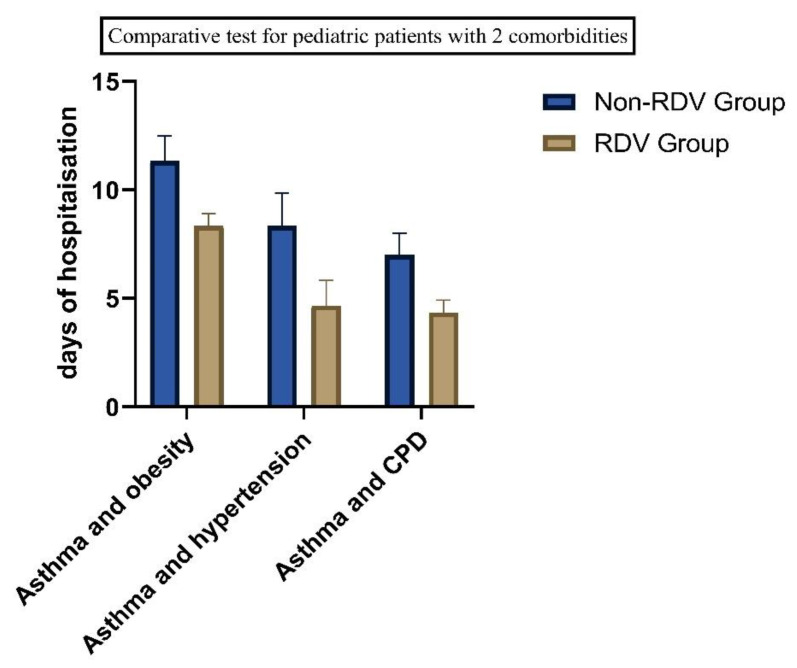
Comparatives test for patients with comorbidities, as of the number of days of hospitalization, we found statistical significance in all groups. (*p* = 0.05).

We found significant differences between the patients with comorbidities. Sixteen patients presented with asthma and obesity, (BMI > 30 kg/m^2^), 13 patients were diagnosed with asthma and hypertension, and eight have asthma and chronic pulmonary disease (See Table 1). Twenty-three patients with one comorbidity received RDV and 25 (19.84%) with two comorbidities were included in the RDV group. Of the 42 children with allergies, 43% had at least one comorbidity condition and 23% had two comorbidities. 27.7% had preexisting chronic pulmonary disease and 21.43% had obesity. Comorbidities were independently associated with increased days of hospitalizations (See Figure 4 and Figure 5).

The demographic data of 42 patients with allergic disease are presented in Table 3. Most of the patients presented polysensitization to different allergens. Monosensitized patients were considered if they had sensitization only to weed pollen. All data were collected from the patient’s medical records from an allergologist specialist.

We performed a series of univariate and multivariate proportional odds ratio models for significant parameters such as body temperature, oxygen saturation, heart, respiratory rate, and the allergies, which were correlated with RDV administrations in the analysis. The adjusted OR showed significant results when including allergies (asthma, atopic dermatitis, and allergic rhinitis) when correlated with fever and respiratory symptoms and gastroenteritis correlated with nausea, vomiting, and diarrhea (*p* < 0.05). 

The values of heart rate, respiratory frequency, cough, and dyspnea in this association between potential risks and RDV administration did not show any significant variation when compared between them. The results are illustrated in Table 4. 

More patients in the RDV group in total had been symptomatic for 2.1 median days, [IQR 1–3], before starting RDV treatment, and a higher proportion of the RDV group had clinical improvement and laboratory improvement results in comparative tests for the mild to moderate in the RDV groups (See Table 5). There was a difference in time to recovery between pediatric patients based on clinical severity subgroups for allergies. Patients treated with RDV reached clinical improvement faster than the non-RDV group (median, 4 days [IQR, 3.0–7.0 days] vs. 7 days [IQR, 4.0–16.0 days]. For the group with moderate disease, we found clinical improvement after 10 days, [IQR 4.0–10.0 days], while after 5 and 7 days of RDV, we found statistical significance between the RDV and non-RDV groups (*p* < 0.05) (See Figure 3). 

**Figure 6:**
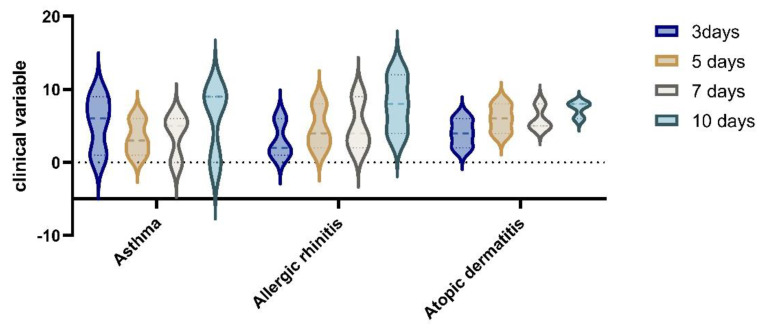
Clinical variables in non−RDV group for patients with allergies.

**Figure 7:**
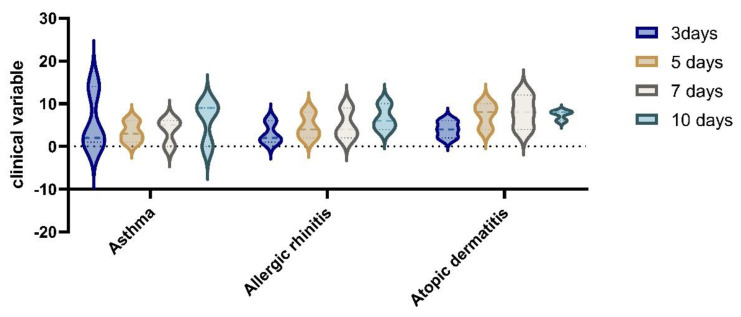
Clinical improvements after RDV administration in the allergies group with asthma, allergic rhinitis, and atopic dermatitis at 3, 5, 7, and 10 days after hospitalization.

In the analysis of clinical variables for patients with allergies, in the group with RDV treatment vs non RDV regimens, we observed an improvement in symptomatology for RDV group. The difference in the clinical variable on day 3 to day 10 of hospitalisation, was statistically significant (*p* = 0.05) by Wilcoxon rank sum test; the proportional odds assumption was met for this comparison). (See Figure 6 and Figure 7).

Moreover, the subgroup analysis, based on treatment for pediatric cases with COVID-19, and the laboratory results indicated that there was a significant difference between the different categories in both groups with RDV and non-RDV (See Table 6).

The laboratory findings show there is a significant relationship between antiviral treatment in COVID-19 patients. Table 6 shows that among the 11 parameters analyzed between the two groups with RDV and non-RDV, and in the sub-groups, ALT, AST, WBC, lymphocyte, CRP, prothrombin time, and LDH were significantly different (*p* < 0.05). No important differences were apparent between the patients who received antibiotics, dexamethasone, and corticosteroids compared with patients who received RDV. Such analysis shows how the laboratory findings changed during the disease progression and whether they acquired a prognostic value. There was no statistical significance in groups in the use of corticosteroids and antibiotics. Of the total study group, 85 (34%) patients received dexamethasone. Additional antibiotic treatments were administered for 105 patients, (42%), as follows: 29 patients received cefotaxime, 34 patients received sulfamethoxazole /trimethoprim, 23 patients received sulbactam/ampicillin, and 19 received vancomycin.

**Figure 8:**
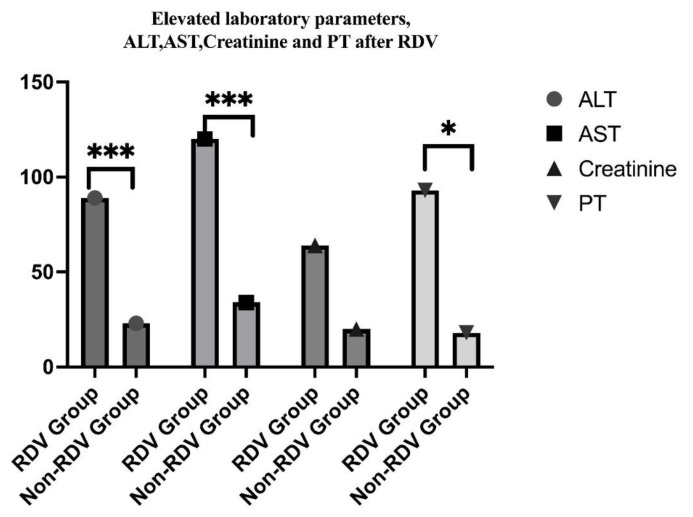
Comparative study on modified laboratory parameters after RDV administration in children, indicating statistical significance in ALT and AST, and prothrombin time (*** *p* = 0.0001, * *p* = 0.01).

The adverse effects, such as increased liver enzymes, occurred in 68 patients who have elevated ALT levels and 78 for AST, in the RDV group vs. 21 and 32 in the non-RDV group, *p* = 0.032 and 0.001) (See Figure 8). Overall, our patients experienced mild adverse reactions to RDV, (grades 1 and 2), which did not lead to discontinuation. No allergic reactions were attributed to RDV in pediatric patients included in this study. Laboratory safety findings also included creatinine increased level, which was higher in the non-RDV group without statistical significance.

We also found a correlation between RDV administration and no asthma exacerbation in the study group (*p* < 0.05). All asthma patients maintained the asthma symptoms and showed an improvement in days of hospitalizations after RDV administration, 8 days, IQR [5,6,7,8,9,10]) than in the non-RDV group (14 days, IQR [8,9,10,11,12,13,14,15,16] and improvements in clinical variables. 

**Figure 9:**
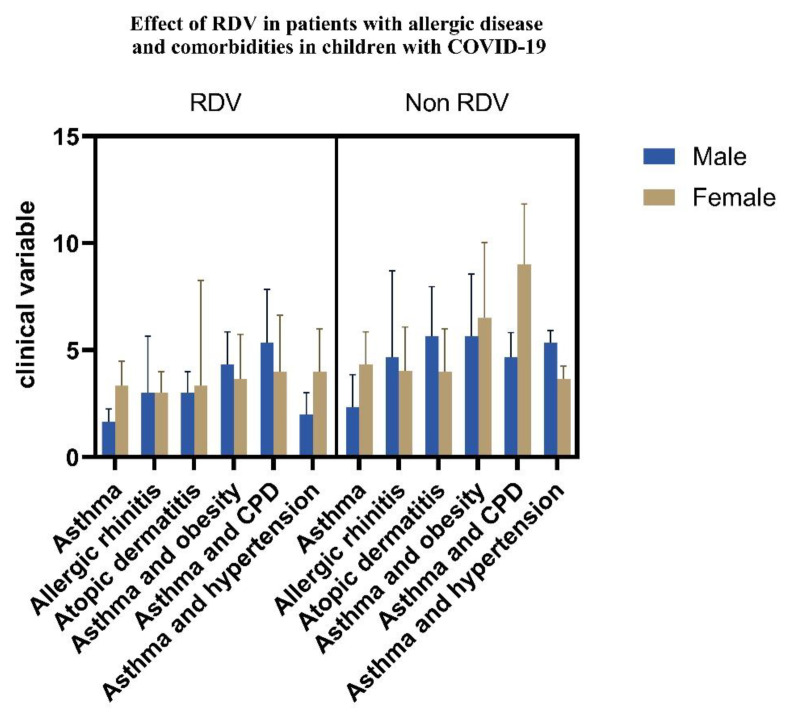
RDV effects in pediatric patients with allergies and comorbidities (*p* < 0.05).

We performed gender analysis of the clinical variables in patients with allergies and groups with comorbidities when RDV was administered (See Figure 9).

All patients in the study group recovered without any sequelae after RDV treatment.

## 4. Discussion

The results of our study suggest that RDV administration in children with SARS-CoV-2 is associated with clinical benefit compared with symptomatic therapy alone in hospitalized patients with mild, moderate, and severe forms of COVID-19 and especially for the group with allergies. In our study, COVID-19 predominated in males and the age group under five years old was predominant. The primary outcome of time to recovery was four days shorter in the RDV treatment groups. Additionally, we found differences in the treatment effects among clinical subgroups of patients with allergies and COVID-19. We found an increased number of patients with digestive manifestations in a group of children under 5 years and this was similar for infants (dehydration syndrome, diarrhea, vomiting, and loss of appetite). In older children and adolescents, the respiratory clinical picture predominates (nasal obstruction, rhinorrhea, cough, dysphagia, odynophagia, dysphonia, and breathing difficulties). Most of the cases also presented systemic symptoms (fever, chills, fatigue, and altered general condition) and were accompanied by highly elevated inflammatory markers. A decrease in inflammatory markers, as well as liver enzymes, was observed after RDV administration, probably due to recovery. 

The current study shows that RDV may be used safely for children and asthmatic pediatric patients with adequate treatment and good control of asthma, who had a lower risk of a severe manifestation of COVID-19. In this study group, all the patients recovered without invasive mechanical ventilation, with no mortality, even in the group with asthma, but we cannot be sure whether only RDV contributed to the recovery. Our asthmatic patients with COVID-19 reported mild and moderate symptoms and no one needed an intensive care unit or intubation. Most of our asthmatic patients had asthma 3 and 4 of the Global Initiative for Asthma (GINA), and their treatment included a moderate dose of corticosteroids (inhaled) and long-acting bronchodilator inhalers (LABA). In this study, we found that wheezing and dyspnea associated with fever were frequent in the group with asthma, and their favorable evolution after RDV administration allowed us to assess the benefits of antiviral medication in this category. There were no differences in terms of spirometry parameters in children with asthma in the RDV and non-RDV groups. According to GINA, those with well-controlled asthma do not appear to be much more at risk of contracting SARS-CoV-2 or developing severe COVID-19, but those who recently required oral corticosteroids for their asthma have a higher risk of dying from COVID-19. To the best of our knowledge, it is the first study to focus on allergic diseases in children with COVID-19 in our country. 

We found some patients who developed mild to moderate liver dysfunction (elevations of transaminase levels), leukocytosis, lymphopenia, and high prothrombin time, but no serious adverse effects were reported. We had no patients with renal dysfunction, and serum creatinine levels were greater than the upper limit of the age-appropriate normal range in the group with RDV and did not necessitate the discontinuation of RDV. Previous clinical studies in children show that RDV may cause liver dysfunction among pediatric patients. [26,27,30]. ALT elevation after RDV administration ranged between 4–33% in different cohort studies, while AST was higher, with elevation ranging between 4–58% [31,32,33,34]. In our study, hepatic dysfunction, which was defined as serum AST and ALT levels more than five times the upper limit of normal, was found in 53.97% (ALT) and 61.9% (AST) and improved spontaneously. Since some reports show that COVID-19 itself can cause liver dysfunction, it is unclear in our cases whether the cause was RDV therapy or COVID-19. The strength of this study is that it includes the largest number of pediatric patients who received RDV in Romania. Few previous studies have reported the safety and efficacy of RDV in children, or for children with comorbidities [34,35]. Different results were observed, and COVID-19 symptoms have improved more quickly due to RDV administration in patients with obesity, asthma, and chronic pulmonary diseases. In our analyses, the use of RDV was the only independent factor significantly decreasing the days of hospitalization among pediatric patients with comorbidities [36]. Liver involvement with elevated transaminases and obesity in children with COVID-19 coincides with other observations in the literature, but the long-term effects of the SARS-CoV-2 virus on the liver, whether from acute COVID-19 or RDV, are yet to be observed.

We provide a comparative study designed for RDV administration in children, especially for the allergy group, where the effects of therapeutic interventions shortened the duration of hospitalization and improved clinical characteristics. These findings are similar to studies reported by the Centers for Disease Control and Prevention through surveillance of children hospitalized with different clinical forms of COVID-19 who are predisposed to severe evolution and moderate illness [37]. Based on our findings, the treatment with RDV reduced the need for oxygen supplements and prevented the progression to severe respiratory distress in children with asthma when compared to another study in the literature. In our analyses, the use of RDV was initiated after three median days of hospitalization and was the only independent factor significantly decreasing the days of hospitalizations among patients with comorbidities, such as obesity, asthma, and chronic pulmonary disease. Patients receiving RDV recovered faster, had better clinical outcomes, and saw no mortality. According to the GINA criteria, asthma is well controlled if there are daytime symptoms no more than twice or less per week; limitation of activities—none; nocturnal symptoms/awakening—none; and the need for reliever/rescue treatment twice or less per week. Asthma is partly controlled if 1–2 of these criteria are present and uncontrolled if 3–4 of these criteria are fulfilled. Therefore, all patients in the study group were classified as having controlled diseases.

The findings in this report are subject to a few limitations. First, COVID-19-associated hospitalizations might have been missed because vaccinated children were grouped with unvaccinated pediatric patients. We faced a small number of vaccinated patients, especially since, in the category of patients with allergies, parents were reluctant to vaccinate their children in our country. This led to a small number of patients vaccinated against COVID-19 and, consequently, the statistical significance was not relevant.

Secondly, the use of antibiotics may be associated with the result of the safety and efficacy of RDV. The third limitation is the relatively short duration of follow-up for these patients (28 days). The fourth limitation is the clinical evolution of patients with asthma without correlation with RDV-associated asthma treatment. 

## 5. Conclusions

In conclusion, this single-center retrospective study shows that the use of RDV for COVID-19 in children led to no serious adverse events in children, no allergies, and reduced days of hospitalizations in patients with comorbidities and improved moderate and severe forms of COVID-19 associated with asthma, allergic rhinitis, and atopic dermatitis. Further studies should be conducted to determine whether RDV is safe and effective for children with COVID-19, especially those with allergies. 

## Figures and Tables

**Table 1:** Demographic and clinical characteristics of 250 children <18 years old with COVID-19.

Variables	Total Patients n = 250 n (%)	RDV Group n = 126 (50.4) n (%)	Non-RDV Group n = 124 (49.6) n (%)	*p*
Demographic				
Age median [IQR]	3 [2,16]	2 [3–12]	4 [3–17]	0.345
0–1	97 (38.8)	52 (41.27)	45 (36.3)	<0.001
2–5	83 (33.2)	46 (36.5)	37 (30)	0.14
6–14	46 (18.4)	18 (14.29)	28 (22.58)	0.334
15–18	24 (9.6)	10 (7.94)	14 (11.3)	0.809
Male Gender	132 (52.8)	66 (52.6)	66 (53.23)	0.5
Symptoms among pediatric patients (subgroup analysis)				
Mild	109 (43.6)	56 (44.4)	53 (42.7)	0.11
** Fever	96 (88.07)	50 (89.28)	46 (86.3)	0.001
Cough	50 (45.87)	26 (46.43)	24 (45.28)	0.0123
Sore throat	45 (41.28)	25 (44.64)	20 (37.74)	0.543
Runny nose	73 (66.97)	33 (58.9)	40 (75,47)	0.675
Fatigue	70 (64.22)	39 (69.64)	31 (58.49)	0.218
Chills	34 (13.6)	23 (41.07)	11 (20.75)	0.600
Wheezing	14 (5.6)	6 (4.76)	8 (6.45)	0.05
Dyspnea	21 (8.4)	9 (7.14)	12 (9.67)	0.05
* Nausea, vomiting, or diarrhea	74 (67.89)	34 (60.71)	40 (75.47)	0.001
** Respiratory symptoms	93 (85.32)	50 (89.29)	43 (81.32)	0.05
Moderate	137 (53.2)	68 (53.97)	69 (55.7)	0.329
Dehydration	68 (49.63)	38 (55.9)	30 (43.49)	0.541
Bronchiolitis	33 (24.09)	21 (30.9)	12 (17.4)	0.302
* Gastroenteritis	71 (51.82)	39 (57.35)	32 (46.4)	0.001
** Pneumonia	50 (36.5)	29 (42.65)	21 (30.4)	0.05
** Asthma	18 (7.2)	10 (7.94)	8 (6.45)	0.05
# Asthma and obesity	16 (6.4)	9 (7.14)	7 (5.65)	0.9
# Asthma and chronic pulmonary disease	8 (3.2)	6 (4.76)	2 (0.8)	0.3
# Asthma and hypertension	13 (5.2)	10 (7.94)	3 (2.42)	0.7
** Atopic dermatitis	11 (4.4)	7 (2.8)	4 (1.6)	0.05
** Allergic rhinitis	11 (4.4)	6 (2.4)	5 (2)	0.05
Severe	4 (1.6)	4 (3.17)	0	0.219
Respiratory failure	2 (0.4)	2 (1.59)	0	0.5
Sepsis	2 (1.2)	2 (1.59)	0	0.1

* represents statistical correlations between clinical manifestations and diseases in children without allergies, ** represents statistical correlations between clinical manifestations and diseases in children with allergies, # represents patients with comorbidities.

**Table 2:** Analysis of comorbidities associated with RDV administration in hospitalized pediatric patients. Obesity, asthma and CPD were found to be significantly associated with RDV administration, as of clinical respiratory improvements, *p* < 0.05. Data are n (%) of patients.

Comorbidities	RDV Group n = 126	non-RDV Group n = 124	Oxygen Requirement In RDV Group	Oxygen Requirement in Non-RDV	*p*-Value
Obesity n (%)	27 (21.43)	19 (15.32)	9 (33.33)	11 (58)	0.001
Hypertension	14 (11.11)	10 (8.06)	4 (28.57)	6 (60)	0.67
Asthma	10 (7.94)	8 (6.45)	4 (40)	5 (62.5)	0.0032
Chronic pulmonary disease (CPD)	35 (27.7)	24 (19.35)	22 (62.9)	19 (79.2)	0.031
# Asthma and chronic pulmonary disease	14 (11.11)	8 (6.45)	9 (7.14)	10 (8.06)	0.3
# Asthma and hypertension	10 (7.94)	5 (4.03)	3 (2.38)	9 (7.26)	0.4
# Asthma and obesity	9 (7.14)	7 (5.65)	5 (3.97)	11 (8.87)	0.9

# represents children with allergies and comorbidities.

**Table 3:** Demographic data, conditions, and medications of allergic pediatric patients were included in our study. Data are expressed as number and (%) and for age as median and IQR. Abbreviations: inhaled corticosteroids (ICS) and long-acting beta-agonists (LABA), according to international guidelines.

Characteristics of Patients with Allergies	Patients, n = 42
Age (years), median [IQR]	8 [6–12]
Gender, n (%)	
Male	22 (52.38)
Personal history of atopy, n (%)	
Atopic dermatitis	10 (23.8)
Allergic rhinitis	9 (21.43)
Familial history of asthma, n (%)	13 (30.95)
Allergic sensitization n, (%)	42 (100)
Polysensitization	23 (54.76%)
Monosensitization	19 (45.24%)
Allergic Rhinitis n, (%)	11 (26.19)
Mild	6 (14.29)
Moderate	4 (9.52)
Severe	1 (2.38)
Level of asthma control before treatment with RDV, n (%)	18 (100)
Controlled	8 (19.05)
Partial controlled	10 (23.8)
Uncontrolled	0 (0)
Treatment of Asthma patients n, (%)	18 (42.86)
ICS/LABA	12 (28.57)
Leukotriene receptor antagonist	14 (33.33)
Long-acting β_2_-agonist	8 (19.05)
Oral corticosteroid	4 (9.52)
Atopic dermatitis	11 (26.19)
Polysensitization	9 (21.43)
Monosensitization	2 (4.76)

**Table 4:** OR-Odds ratios (95% CI) of multivariate associations between significant parameters and RDV administration among children with allergies who tested positive for SARS-CoV-2, hospitalized between July 2020 to September 2022. The multivariate model was adjusted for disease severity, for patients with asthma, allergic rhinitis, and atopic dermatitis.

Significant Parameters	Unadjusted OR (95% CI) RDV Group	Unadjusted OR (95% CI) Non-RDV	*p*-Value	Adjusted OR (95% CI) RDV Group with Allergies	Adjusted OR (95%CI) Non-RDV with Allergies	*p*-Value
Oxygen saturation	2.26 (1.94–2.48)	2.95 (1.96–3.12)	0.021	1.12 (0.91–1.96)	1.01 (1.65–1.84)	0.001
Heart rate	2.34 (1.35–1.50)	4.64 (2.62–6.45)	0.222	1.24 (1.01–2.94)	1.95 (1.69–2.80)	0.2
Respiratory frequency	3.14 (2.02–4.03)	3.26 (1.95–4.23)	0.122	2.04 (1.86–2.21)	2.92 (1.62–2.03)	0.5
Fever (>38.5 °C)	2.02 (1.92–3.03)	1.12 (0.61–3.89)	0.05	0.62 (0.42–1.26)	0.61 (1.04–1.24)	0.001
Cough	2.29 (1.00–4.60)	2.14 (1.89–4.21)	0.132	1,87 (1.49–3.72)	3.12 (2.92–4.74)	0.131
Dyspnea	2.39 (1.29–3.48)	2.27 (1.14–3.23)	0.21	0.71 (16–2.12)	0.62 (0.84–1.18)	0.05

**Table 5:** Clinical patient characteristics in days of improvements, with and without RDV, in group with allergies.

Parameters	1. Non-RDV Group n = 124	2. RDV Group n = 126	3. Allergies Group RDV n = 23	3` Allergies Group Non-RDV n = 19	4. RDV-Group Mild Form n = 56	5. RDV-Group Moderate Form, n = 68	6. RDV-Group Severe Form, n = 2	*p*-Value
3 days after baseline, n (%)	31 (25)	27 (21.43)	3 (13.04)	2 (10.53)	18 (32.14)	9 (13.24)	0	0.121
5 days after baseline, n (%)	23 (18.55)	43 (34.13)	7 (30.43)	5 (26.32)	19 (33.93)	24 (35.3)	0	0.0065 *^,^ ***
7 days after baseline, n (%)	33 (26.61)	30 (23.81)	9 (39.13)	5 (26.32)	10 (17.86)	20 (25)	0	0.0137 *^,^ ***
10 days after baseline, n (%)	37 (29.84)	26 (20.63)	4 (17.4)	7 (36.84)	8 (14.29)	18 (26.47)	2 (100)	0.001 **

* *p*-value * for column 1 vs. 2, ** for 4 vs. 5 columns, *** for 3 vs. 3` columns.

**Table 6:** The role of antiviral RDV, antibiotics, and dexamethasone in laboratory changes in COVID-19 for pediatric patients included in this study. Such analysis shows how the laboratory findings changed during the COVID-19 clinical form.

Parameters	1.Non-RDV Group n = 124	2.RDV Group n = 126	3.RDV-Group Mild Form n = 56	4.RDV-Group Moderate Form, n = 68	5.RDV-Group Severe Form, n = 2	*p*-Value
Treatment						
RDV	-	126	56	68	2	0.001 *^,^**
Dexamethasone, n (%)	46(37.1)	39(31)	20(53.71)	17(25)	2(100)	0.45
RDV and Dexamethasone, n (%)	-	62(49.2)	31(55.36)	29(42.65)	2(100)	0.11
Antibiotics, n (%)	33(26.6)	72(57.14)	29(51.8)	41(60.3)	2(100)	0.112
Laboratory n (%)						
AST, U/L > 5xnormal values.	23(18.55)	76(60.32)	38(67.86)	36(53)	2(100)	0.05 *^,^**
ALT, U/L > 5xnormal values,	22(17.74)	70 (55.5)	36(64.3)	32(47.1)	2(100)	0.001 *^,^**
Total bilirubin, increased, g/dL	18(14.5)	34(27)	21(37.5)	12(17.6)	1(50)	0.32
Anemia	11(8.87)	37(29.4)	14(25)	21(30.9)	2(100)	0.01
Leukocytosis	59(47.58)	76(60.32)	44(78.6)	30(44.12)	2(100)	0.02
Lymphopenia	34(27.42)	51(40.5)	28(50)	22(32.35)	1(50)	0.01 *
Neutrophilia	23(18.55)	55(43.65)	32(57.14)	21(30.88)	2(100)	0.21
Thrombocytosis	22(17.74)	61(48.4)	31(55.36)	28(41.18)	2(100)	0.11
High Prothrombin time	21(16.94)	70(55.5)	32(57.14)	36(53)	2(100)	0.03
LDH elevated.	27(21.77)	75(59.52)	42(75)	31(45.6)	2(100)	0.001 *
CRP elevated	25(20.16)	76(60.32)	35(62.5)	39(57.35)	2(100)	0.05 *

* column 1 vs. 2, ** column 3 vs. 4.

## Data Availability

Data is unavailable.
